# Feline Stool-Associated Circular DNA Virus (FeSCV) in Diarrheic Cats in China

**DOI:** 10.3389/fvets.2021.694089

**Published:** 2021-06-16

**Authors:** Xiangqi Hao, Yanchao Li, Xinkai Hu, Xueying Fu, Jie Dong, Haoyang Zhang, Pei Zhou, Shoujun Li

**Affiliations:** ^1^College of Veterinary Medicine, South China Agricultural University, Guangzhou, China; ^2^Guangdong Provincial Key Laboratory of Prevention and Control for Severe Clinical Animal Diseases, Guangzhou, China; ^3^Guangdong Provincial Pet Engineering Technology Research Center, Guangzhou, China

**Keywords:** feline stool-associated circular DNA virus (FeSCV), cats, China, genetic characterization, diarrheic

## Abstract

Feline stool-associated circular DNA virus (FeSCV) is an unclassified circular replication-associated protein-encoding single-stranded (CRESS) DNA virus that was discovered in cats in Japan in 2018. Few studies on the genomic characteristics and prevalence of FeSCV have been conducted. To investigate whether FeSCV has been circulating in domestic cats in Guangdong, China, fecal samples were collected from cats with diarrhea in an animal hospital in 2018 to promote research on FeSCV. The FeSCV genome was obtained by PCR amplification and sequencing, and the detected virus was named PY4 (GenBank No. MT732515). The genome of PY4 was 2,034 nt in size, which was 12 nt smaller than the reported genome of Japanese FeSCV strains (KU7, KU8, KU9, KU14) (2,046 nt). The PY4 strain shared 95.1 ~ 95.5% homology with Japanese FeSCV strains. Notably, the Cap protein of PY4 was mutated at 15 amino acid sites, and the PY4 genome contained a unique open reading frame 3. In addition, there were two additional base insertions in the stem-loop structure of PY4, and the nucleotide homology of the spacer region was not high. A phylogenetic tree based on Rep proteins showed that PY4, Japanese FeSCVs and rodent stool-associated circular viruses (RodSCVs) clustered together, suggesting that they might share a similar origin in their phylogenetic evolution. In this study, samples collected in Guangzhou, China, in 2018 were subjected to an etiological investigation, and 20% (2/10) of the samples were positive for FeSCV. The ORFs, stem-loop structures, Cap proteins and intergenic region sequences of PY4 were significantly different from those reported in Japan. This is the first report of FeSCV in domestic cats with diarrhea in China, and further epidemiological studies are urgently needed to assess the impact of the virus on cats.

## Introduction

Recently, many viruses of the *Circoviridae* family have been found in different animal tissues, feces and even in human spinal fluid (1, 2). Notably, Cycloviron-Vietnam has been detected in both humans and animals (3). With the discovery of novel circular replication-associated protein-encoding single-stranded (CRESS) DNA viruses, the known range of Circoviridae members has been expanded. Feline stool-associated circular virus (FeSCV), a member of the Circoviridae family with a circular genome (2,046 nt) encoding two open reading frames (ORFs), was found in the feces of diarrheal cats in Japan in 2018 (4). This novel virus was suspected of being associated with enteric disease (4). Members of Circoviridae may cause immunosuppression, increasing the chance of the host being infected other pathogens (5, 6), so the novel FeSCV is worthy of further study. To date, the molecular characterization of FeSCV has been reported only in Japan. Moreover, four reported FeSCV strains show high genomic homology. No further studies on this newly identified CRESS DNA virus have been reported in other countries, and it is worth obtaining more information about FeSCV.

## Materials and Methods

### Clinical Samples

From August to November 2018, fecal samples were collected from cats at an animal hospital in Guangdong, China. All samples were obtained from cats suffering severe diarrhea. Each sample was collected with the oral permission of the cat owner, and the feces were collected without causing any pain to the animals. All collected samples were stored at −80°C until analysis.

### Nucleic Acid Extraction and Reverse Transcription

DNA/RNA was extracted using the SimplyP Virus DNA/RNA Extraction Kit (BSC67S1, Bioer Technology, China) according to the manufacturer's instructions. In addition to the detection of FeSCV, the presence of other viruses causing intestinal diseases, including feline panleukopenia virus (FPV), feline kobuvirus (FeKoV), feline astrovirus (FeAstV) and feline enteric coronavirus (FeCoV), was also assessed. DNA/RNA was directly used for the detection of DNA viruses, including FeSCV and FPV. The RNA sequences of FeKoV, FeAstV and FeCoV were reverse transcribed (RT) using 5 × All-In-One RT Master Mix (G490, Applied Biological Materials, Canada) to synthesize first-strand cDNA.

### PCR Detection of FeSCV and Other Feline Enteric Pathogens

First, we detected FeSCV by referring to the primers reported in previous research (FeSCV1F 5′-GCTAAGGTCTGCCTCAGGTG and FeSCV1R 5′-CTATGTCCAGGTCGGGAGAA). The amplification procedure was as follows: 95°C for 5 min, 35 cycles of 95°C for 30 s, 55°C for 45 s, and 72°C for 45 s, and a final extension at 72°C for 7 min; the amplified product of the positive sample was 303 bp (4). Then, for the detection of other feline viruses described above, the primer sequences used in this study came from a multiplex PCR detection kit for several feline pathogens that was invented in our laboratory (7). All the primers were synthesized at Beijing Liuhe Huada Gene Technology Company (Beijing, China). The procedure for the detection of multiple pathogens was as follows: initial denaturation at 98°C for 3 min, 36 cycles of 98°C for 20 s, 55.6°C for 1 min, and 72°C for 1 min, and a final extension at 72°C for 5 min. Ex-taq Master Mix (RR902A, Takara, Japan) was used for PCR amplification, followed by electrophoresis in a 1.2% agarose gel. Positive virus samples were determined by observing the size of product bands and then excising them from the gel to send for sequencing.

### Genome Amplification and Sequencing

First, Phi29 DNA Polymerase (LP101-01, Transgen, China) was used to amplify the single-stranded circular genome of FeSCV from the extracted DNA. The steps for genomic PCR amplification were performed according to a previous study conducted in Japan (4). Different fragments were amplified using Premix Prime STAR HS DNA polymerase (Takara, Beijing, China). After A bases were added, the 303 and 1,800 bp bands were cloned into the pMD-19T simple vector, and the ligation products were transformed into DH5α cells. After the monoclonal bacteria were tested by PCR, the positive clones were sent to Beijing Liuhe Huada Gene Technology Company (Beijing, China) for sequencing. Finally, by splicing the 303 and 1,800 bp bands, genome information was obtained.

### Characterization of the FeSCV Genome

First, SnapGene software (version 4.2.4; from Insightful Science; available at snapgene.com) was used to splice the genome sequences together and draw the complete gene map. The DNASTAR package (DNASTAR, Madison, WI, USA) was used for sequence alignment and homology analysis. The NCBI Open Reading Frame (ORF) Finder (https://www.ncbi.nlm.nih.gov/orffinder/) was used to identify the putative main ORFs of FeSCV. The stem-loop structure of FeSCV was verified using the Mfold web server (http://mfold.rna.albany.edu/?q=mfold) (8).

### Identification of Replication Initiator Protein (Rep) and Capsid Protein (Cap) Mutations

The Rep protein is a non-structural protein encoded by the Rep gene that shows relatively high amino acid conservation. Among the members of the *Circovirus* genus, the Rep gene is necessary for viral replication (9). In general, the Cap protein is the main protective antigen protein of the virus and determines its antigenicity. Additionally, the Cap protein is related to the virulence of the virus, and it also interacts with host proteins (10–12). In this study, we analyzed the amino acid sites where mutations occurred in the Rep and Cap proteins, and BioEdit (version 7.0.9.0) was used for amino acid mutation analysis (13).

### Phylogenetic Analysis of the Rep Gene

To understand the genetic evolution of the FeSCV strain obtained in this study, the Rep amino acid sequence of the new virus was compared with those of control strains including FeSCV-KU7, FeSCV-KU8, FeSCV-KU9, and FeSCV-KU14 (4), and 43 representative Circovirus, Smacovirus, Cyclovirus and Unclassified CRESS DNA virus strains. Smacovirus was selected as an outgroup to determine the roots of the evolutionary tree. Finally, the neighbor-joining tree (p-distance model) (bootstrap value = 1,000) was built using Mega software (Version: 7.0.26) (14).

## Results

### Collection and Detection of Fecal Samples

From August to November 2018, a total of 10 samples were obtained from 10 diarrheic cats at an animal hospital in Guangdong. After PCR and RT-PCR detection, the results showed that 20% of the samples (2/10) were positive for FeSCV and that cats were coinfected with other intestinal viruses (Table 1). All samples producing target bands were verified by sequencing the PCR products. FPV, FeKoV, and FeAstV showed high detection rates in this study.

**Table 1:** Results of multiple virus tests on cat fecal samples.

**Sample NO**.	**FeSCV**	**FPV**	**FeKoV**	**FeAstV**	**FeCoV**
1	−	+	+	+	−
2	−	+	+	+	+
3	−	+	+	+	−
4	+	+	+	+	−
5	−	+	+	+	−
6	+	+	+	+	+
7	−	+	+	+	−
8	−	+	+	−	+
9	−	+	+	+	−
10	−	+	+	+	−

*+ means the result was positive for target viruses and − means the result was negative for target viruses*.

### Genome Sequence and Characterization Analysis

**Figure 1:**
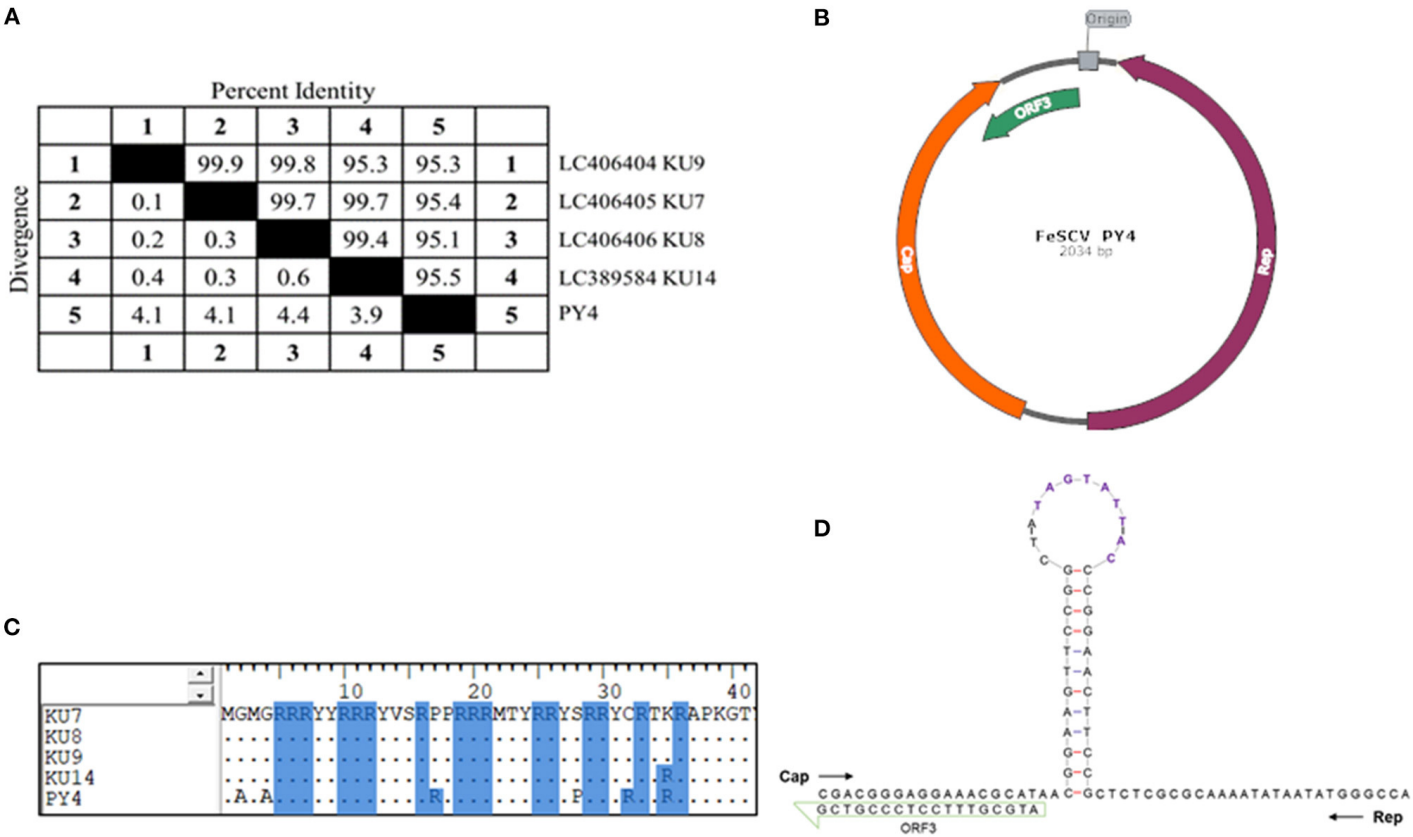
The genomic characteristics of FeSCV-PY4. **(A)** Whole-genome homology analysis of PY4 with other Japanese isolates. **(B)** The genome map of the PY4 strain. Cap is indicated by an orange arrow, Rep by a purple arrow and ORF3 by a green arrow. **(C)** Amino acid analysis of the FeSCV N-terminus. Arginine residues are highlighted with a blue background. **(D)** The stem-loop structure of the PY4 strain. The conserved canonical non-anucleotide motif (5′ TAGTATTAC) is shown in purple.

In the course of the etiological investigation, one sample was accidentally degraded during storage, and another sample was selected, from which the genome of FeSCV was amplified to study its characteristics. Phi29 DNA polymerase was found to be effective for the downstream PCR amplification of the extracted genomes. After sequencing several monoclonal colonies, genome information was obtained. The FeSCV strain obtained in this study was named PY4 (GenBank number: MT732515). The genome of the PY4 strain was significantly different from that of the 4 strains reported in Japan. The genome of PY4 was 2,034 nt in size, which was 12 nt smaller than the reported genome of the FeSCV strain (KU7, KU8, KU9, KU14) from Japan (2,046 nt). The content of AT bases in the PY4 genome was 41.99% and that of CG bases was 58.01%. Homology analysis by using Meg Align software revealed that the PY4 strain showed homology of 95.1~95.5% with the FeSCV-KU7, FeSCV-KU8, FeSCV-KU9, and FeSCV-KU14 strains (Figure 1A).

Two main ORFs, encoding the Rep and Cap proteins, were identified with NCBI-ORF Finder, as reported in a previous study. Unexpectedly, a third major ORF in addition to those of Rep and Cap was found in the PY4 strain (Figure 1B), which differed from the FeSCV-KU14 strain.

The Rep gene (ORF1) was located (56 nt ~ 1,015 nt) on the complementary strand and encoded a 319 aa protein. The Cap gene (ORF2) was located (1,134 nt ~ 1,878 nt) on the sense strand and encoded a 246 aa protein. ORF3 (1,775 nt ~ 2,017 nt) and ORF1 were both located on the complementary chain. The unique ORF3 gene encoded an 80 aa protein whose function is unknown. The Rep protein of the PY4 strain showed amino acid homology of 99.4% ~ 99.7% with those of the KU7, KU8, KU9, and KU14 strains. Similarly, only rolling-circle replication (RCR) motif III was present in PY4 (15), while the Walker A motif of the SH3 helicase in the Rep protein of PY4 had the sequence GPSGSGKS. The homology of the Cap protein was low (91.5% ~ 93.5%). There were many duplicated arginines in the N-terminus of the Cap protein, making it highly alkaline. Analysis by BioEdit alignment suggested that the N-terminus of the Cap protein in the PY4 strain contained more arginine residues than those of Japanese FeSCVs (Figure 1C), which may be involved in its nuclear localization.

The stem-loop structure of the virus is very important for its replication. In our study, a conserved canonical non-anucleotide motif (5′ TAGTATTAC) was found in the large intergenic region (LIR) of PY4. Next, the Mfold Web server was used to predict the stem-loop structure of the virus. However, there were differences between the stem-loop structure of the PY4 strain and the KU14 strain. Interestingly, additional insertions of T and A bases were found in the loop structure of the PY4 strain (Figure 1D), which are worthy of further study to determine whether they affect virus replication. LIR and small intergenic region (SIR) sequences were also non-conserved between PY4 and the KU14 strain. The nucleotide homology of LIR between the PY4 and KU14 strains was 80.4%, and the nucleotide homology of SIR between them was 86.9%. These results suggested that there were significant differences in the stem-loop structure and intergenic region sequences among the strains from different regions.

### Mutations of Rep and Cap Genes Identified in This Study

The Rep protein is necessary for viral replication, and the Cap protein usually determines antigenicity, interacts with host proteins and is also related to virulence. Therefore, we analyzed mutations in two major proteins in the PY4 strain, which can provide the basis for further study of the pathogenicity of the virus. Amino acid sequence alignment analysis showed that there was only one mutation in the Rep protein, at site 54, resulting in a V to I substitution. The Cap protein showed many mutation sites compared with previously reported Cap sequences. Compared with KU14 and the other three strains, PY4 showed 15 novel and unique mutant amino acid sites (Supplementary Figure 1). According to the above comparison results, we speculated that PY4 and KU14 showed little difference in replicative ability, but PY4 might belong to a new antigenicity strain branch.

### Rep Gene Phylogenetic Analysis

**Figure 2:**
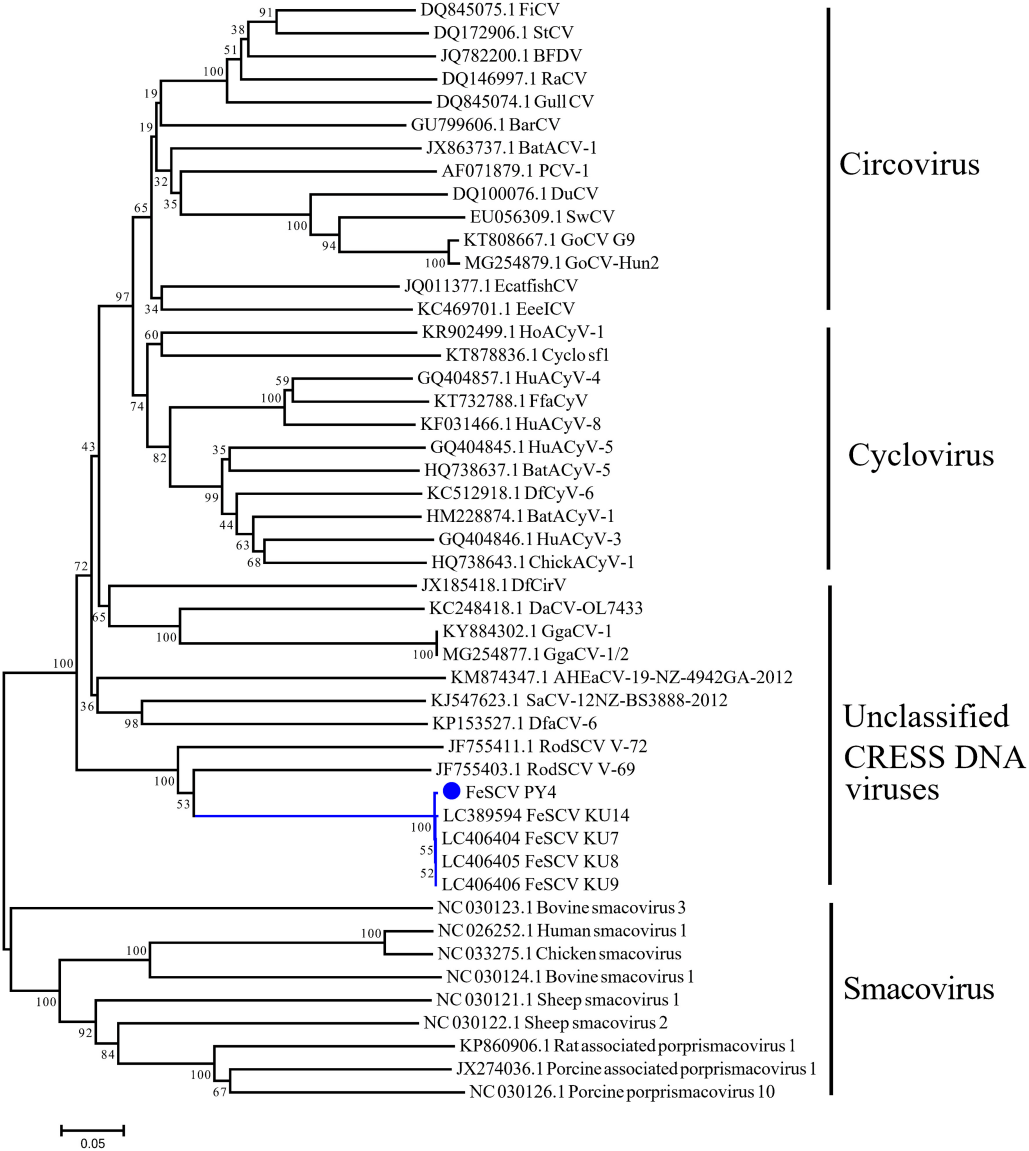
Neighbor-joining tree based on the Rep amino acid sequences of CRESS DNA viruses. The tree was constructed using the neighbor-joining method carried out in MEGA 7.0.26 with bootstrap tests of 1,000 replicates (p-distance model). The blue dot indicates the virus identified in this study.

To further understand the phylogeny of Chinese FeSCV-PY4, we constructed a neighbor-joining tree (p-distance model) (bootstrap value = 1,000) based on the Rep amino acid sequences of FeSCV and the reference strains. The evolutionary tree showed that PY4 was closely related to the Japanese FeSCV strains and shared an evolutionary branch with rodent stool-associated circular viruses (RodSCVs), suggesting that they might have a similar origin in phylogenetic evolution (Figure 2). Although both RodSCV and FeSCV belonged to unclassified CRESS DNA viruses, the bootstrap value between the unclassified CRESS DNA viruses and other viruses was 100, indicating that these newly discovered viruses show some similarities with the previously studied viruses, which is worthy of further study.

## Discussion

In recent years, with the development of sequencing technology, an increasing number of novel CRESS DNA viruses have been discovered. Although the number of CRESS DNA viruses is large, relatively few studies of these viruses have been carried out (16, 17). Among mammalian circoviruses, PCV-2 alone seems to be the focus of significant research, and with this virus causes miscarriage in sows and death in piglets, seriously impacting the development of the pig industry (18). Canine circovirus and mink circovirus have been discovered recently, and research on detection methods for these viruses and their pathogenesis is gradually being carried out (19–22). The CRESS DNA viruses have a wide range of hosts and have been found in birds, rodents, chimpanzees, and even humans (1, 23–25). At present, little progress has been made in determining whether CRESS DNA causes disease. One study suggested that CRESS DNA without apparent pathogenicity may contribute to the development of infectious diseases by slightly affecting host immunity and facilitating the invasion of the host by other pathogens (26).

FeSCV is a virus that was newly reported in 2018 in Japan and was thought to cause diarrhea in cats. However, its genetic characteristics are poorly described. In this study, feline stool samples were collected in Guangdong, China. The detection results suggested that coinfection with multiple viruses in diarrheal cats was common, and two samples were positive for FeSCV. One strain (named PY4) was selected and amplified to determine its genome. It should be noted that FeSCV is a single-stranded DNA virus and the gene of this virus was found to be unstable and easy to degrade during our study. Therefore, after positive samples were detected, the samples can be packaged and stored at −80°C to avoid repeated freezing and thawing. In addition, Phi29 DNA Polymerase treatment will be helpful for genomic signal amplification and avoiding the problems associated with DNA degradation. The genome of PY4 was 2,034 nt in size, which was 12 nt smaller than the reported genome of the FeSCV strains from Japan. Homology analysis suggested that the PY4 strain shared homology of 95.1%~ 95.5% with the FeSCV-KU7, FeSCV-KU8, FeSCV-KU9, and FeSCV-KU14 strains. ORF finder predicted that there were three main ORFs in PY4. This finding differs from a previous report in Japan because although we looked within all 4 strains of FeSCV from Japan, we did not find the same ORF3 found in PY4. We hypothesize that the presence or absence of ORF3 is primarily caused by genetic mutations. Small ORFs may often be involved in virulence processes such as apoptosis, which is reflected in ORF3 of PCV2 (27–29). Therefore, the function of ORF3 in FeSCV will be further studied in the future.

In the comparison of the stem-loop structures of the PY4 strain and the reported Japanese FeSCV strains, additional insertions of T and A bases were found in the loop structure of the PY4 strain. Through the comparison of SIR and LIR sequences between different strains, we found that the sequence homology of intergenic regions between PY4 and KU14 was not high. The genome structures of FeSCVs differed significantly between China and Japan, which may be due to geographical isolation with independent evolution. Whether these different structures contribute to the different pathogenicities of the viruses remains to be further studied.

The two most important proteins of FeSCV, Rep and Cap, are related to replication and antigenicity, respectively. By comparing the amino acid sequences of these proteins, we found that there were 15 unique amino mutations in PY4 compared with the Cap proteins of four reference strains, which may have arisen during long-term evolution after geographical isolation. However, only one amino acid site was mutated in the Rep protein. In conclusion, the antigenicity of the PY4 strain may be different from that of the reported strains.

The Rep gene is believed to be the only phylogenetic marker of the CRESS DNA virus. In this study, a phylogenetic tree based on the Rep protein was constructed using the neighbor-joining method, and the results showed that the PY4 and KU14 strains shared a common ancestor. It is worth noting that the bootstrap value between unclassified CRESS DNA viruses and other viruses was 100, indicating that these newly discovered viruses shared common characteristics with the previously studied viruses. Recently, it has been suggested that parasites may be potential hosts for CRESS DNA viruses; however, the parasite genomes did not actually contain CRESS endogenous viral elements (30). Coincidentally, a sequence of *Giardia intestinalis* was indeed found in the FeSCV genome. According to previous studies, *Giardia intestinalis* has not been detected in cats with FeSCV-positive samples (4); therefore, FeSCV cannot be considered the direct origin of *Giardia intestinalis*. Because CRESS DNA viruses are prone to genetic mutation or recombination, we speculated that FeSCV may infect a host simultaneously with *Giardia intestinalis* at a certain time and thereby obtain exogenous genes through gene recombination. As a result, the versatility of the FeSCV genome would have been enriched, and the exogenous genes would have been preserved during evolution. The origin and evolution of FeSCV may be complex, so studies on its natural hosts and pathogenicity are urgently needed.

In summary, this investigation provides molecular information on FeSCV from China. The genomic characteristics of PY4 were significantly different from those of FeSCV strains from Japan, which may be due to geographical isolation and independent evolution. Our study combined with previous reports from Japan showed that FeSCV has a high prevalence rate and shows variation in its virus genes. We speculate that FeSCV is related to diarrhea in cats. Unfortunately, the isolation of the virus is difficult due to the presence of other viruses in FeSCV-positive samples. As the genome of FeSCV is small, there is an opportunity to establish an infectious cloning method to investigate whether the virus is pathogenic in the future.

Because the trade of cats among different countries is common and the monitoring of FeSCV has not yet been carried out, more research should proceed to determine whether FeSCV causes severe diarrhea and to determine the prevalence of FeSCV in other regions. Global surveillance should also be strengthened to investigate how FeSCV is spread among cat populations. These efforts are critical for animal health and indeed for public health. In conclusion, we have described the first identification and full genomic characterization of FeSCV in cats in China, which will provide fundamental knowledge about FeSCV strains circulating in China.

## Data Availability Statement

The data presented in the study are deposited in GeneBank of NCBI repository, accession number (MT732515).

## Ethics Statement

The study was reviewed and approved by South China Agricultural University Experimental Animal Welfare Ethics Committee.

## Author Contributions

XH: conceptualization, methodology, investigation, visualization, formal analysis, and writing—original draft preparation. YL and XH: methodology and investigation. XF, JD, and HZ: investigation, sample collection, and preservation. SL and PZ: conceptualization, writing—review and editing, funding acquisition, project administration, and supervision. All authors contributed to the article and approved the submitted version.

## Conflict of Interest

The authors declare that the research was conducted in the absence of any commercial or financial relationships that could be construed as a potential conflict of interest.

## Supplementary Material

Supplementary Figure 1 Amino acid map of the FeSCV Cap protein. The ClustalW Multiole alignment method was used to analyze amino acid mutations in BioEdit (version 7.0.9.0).

## References

[1] Cui L Wu B Zhu X Guo X Ge Y Zhao K. Identification and genetic characterization of a novel circular single-stranded DNA virus in a human upper respiratory tract sample. Arch Virol. (2017) 162:3305–12. 10.1007/s00705-017-3481-3 28707271

[2] Liu Q Wang H Ling Y Yang SX Wang XC Zhou R. Viral metagenomics revealed diverse CRESS-DNA virus genomes in faeces of forest musk deer. Virol J. (2020) 17:61. 10.1186/s12985-020-01332-y 32334626 PMC7183601

[3] Tan LV van Doorn HR Nghia HD Chau TT Tu LT de Vries M. Identification of a new cyclovirus in cerebrospinal fluid of patients with acute central nervous system infections. Mbio. (2013) 4:e213–31. 10.1128/mBio.00231-13 23781068 PMC3684831

[4] Takano T Yanai Y Hiramatsu K Doki T Hohdatsu T. Novel single-stranded, circular DNA virus identified in cats in Japan. Arch Virol. (2018) 163:3389–93. 10.1007/s00705-018-4020-6 30218220 PMC7087140

[5] Li P Zhang Z Jia R Mao S Wang M Jia R. Rescue of a duck circovirus from an infectious DNA clone in ducklings. Virol J. (2015) 12:82. 10.1186/s12985-015-0312-6 26025466 PMC4450480

[6] Lunney JK Fang Y Ladinig A Chen N Li Y Rowland B. Porcine reproductive and respiratory syndrome virus (prrsv): pathogenesis and interaction with the immune System. Annu Rev Anim Biosci. (2016) 4:129–54. 10.1146/annurev-animal-022114-111025 26646630

[7] Zhou P Xiao X Hao X Li S. mPCR Primer Composition for Simultaneously Detecting Multiple Cat Intestinal Pathogens, and Kit There of 2020. Patent no. 2020.8.7. Chinese Patent, Guangzhou (2020). Available online at: http://www2.soopat.com/Patent/202010266277

[8] Zuker M. Mfold web server for nucleic acid folding and hybridization prediction. Nucleic Acids Res. (2003) 31:3406–15. 10.1093/nar/gkg595 12824337 PMC169194

[9] Mankertz A Caliskan R Hattermann K Hillenbrand B Kurzendoerfer P Mueller B. Molecular biology of Porcine circovirus: analyses of gene expression and viral replication. Vet Microbiol. (2004) 98:81–8. 10.1016/j.vetmic.2003.10.014 14741119

[10] Huang LP Lu YH Wei YW Guo LJ Liu CM. Identification of one critical amino acid that determines a conformational neutralizing epitope in the capsid protein of porcine circovirus type 2. Bmc Microbiol. (2011) 11:188. 10.1186/1471-2180-11-188 21859462 PMC3224128

[11] Gu J Cao R Zhang Y Lian X Ishag H Chen P. Deletion of the single putative N-glycosylation site of the porcine circovirus type 2 Cap protein enhances specific immune responses by DNA immunisation in mice. Vet J. (2012) 192:385–9. 10.1016/j.tvjl.2011.08.005 22015141

[12] Lekcharoensuk P Morozov I Paul PS Thangthumniyom N Wajjawalku W Meng XJ. Epitope mapping of the major capsid protein of type 2 porcine circovirus (PCV2) by using chimeric PCV1 and PCV2. J Virol. (2004) 78:8135–45. 10.1128/JVI.78.15.8135-8145.2004 15254185 PMC446101

[13] Hall T. BioEdit: An important software for molecular biology. GERF Bull. Biosci. (2011) 2:60–1.

[14] Kumar S Stecher G Tamura K. MEGA7: Molecular Evolutionary Genetics Analysis Version 7.0 for Bigger Datasets. Mol Biol Evol. (2016) 33:1870–4. 10.1093/molbev/msw054 27004904 PMC8210823

[15] Rosario K Breitbart M Harrach B Segales J Delwart E Biagini P. Revisiting the taxonomy of the family Circoviridae: establishment of the genus Cyclovirus and removal of the genus Gyrovirus. Arch Virol. (2017) 162:1447–63. 10.1007/s00705-017-3247-y 28155197

[16] Malathi VG Renuka DP. ssDNA viruses: key players in global virome. Virusdisease. (2019) 30:3–12. 10.1007/s13337-019-00519-4 31143827 PMC6517461

[17] Zhao L Rosario K Breitbart M Duffy S. Eukaryotic Circular Rep-Encoding Single-Stranded DNA (CRESS DNA) viruses: ubiquitous viruses with small genomes and a diverse host range. Adv Virus Res. (2019) 103:71–133. 10.1016/bs.aivir.2018.10.001 30635078

[18] Ouyang T Zhang X Liu X Ren L. Co-infection of swine with porcine circovirus type 2 and other swine viruses. Viruses. (2019) 11:185. 10.3390/v11020185 30795620 PMC6410029

[19] Kapoor A Dubovi EJ Henriquez-Rivera JA Lipkin WI. Complete genome sequence of the first canine circovirus. J Virol. (2012) 86:7018. 10.1128/JVI.00791-12 22628401 PMC3393582

[20] Hao X Liu R He Y Xiao X Xiao W Zheng Q. Multiplex PCR methods for detection of several viruses associated with canine respiratory and enteric diseases. PLoS ONE. (2019) 14:e213295. 10.1371/journal.pone.0213295 30830947 PMC6398926

[21] Wang Z Shi Y Wang Y Zhao L Cui X Wen S. Detection of antibodies against canine circovirus in naturally and experimentally infected canines by recombinant capsid enzyme-linked immunosorbent assay. Front Vet Sci. (2020) 7:294. 10.3389/fvets.2020.00294 32548131 PMC7270207

[22] Ge J Gu S Cui X Zhao L Ma D Shi Y. Genomic characterization of circoviruses associated with acute gastroenteritis in minks in northeastern China. Arch Virol. (2018) 163:2727–35. 10.1007/s00705-018-3908-5 29948383 PMC7087342

[23] Phan TG Kapusinszky B Wang C Rose RK Lipton HL Delwart EL. The fecal viral flora of wild rodents. Plos Pathog. (2011) 7:e1002218. 10.1371/journal.ppat.1002218 21909269 PMC3164639

[24] Li L Kapoor A Slikas B Bamidele OS Wang C Shaukat S. Multiple diverse circoviruses infect farm animals and are commonly found in human and chimpanzee feces. J Virol. (2010) 84:1674–82. 10.1128/JVI.02109-09 20007276 PMC2812408

[25] Lima DA Cibulski SP Finkler F Teixeira TF Varela A Cerva C. Faecal virome of healthy chickens reveals a large diversity of the eukaryote viral community, including novel circular ssDNA viruses. J Gen Virol. (2017) 98:690–703. 10.1099/jgv.0.000711 28100302

[26] Shulman LM Davidson I. Viruses with circular single-stranded dna genomes are everywhere!. Annu Rev Virol. (2017) 4:159–80. 10.1146/annurev-virology-101416-041953 28715975

[27] Liu J Zhu Y Chen I Lau J He F Lau A. The ORF3 protein of porcine circovirus type 2 interacts with porcine ubiquitin E3 ligase Pirh2 and facilitates p53 expression in viral infection. J Virol. (2007) 81:9560–7. 10.1128/JVI.00681-07 17581998 PMC1951394

[28] Karuppannan AK Jong MH Lee SH Zhu Y Selvaraj M Lau J. Attenuation of porcine circovirus 2 in SPF piglets by abrogation of ORF3 function. Virology. (2009) 383:338–47. 10.1016/j.virol.2008.10.024 19012942

[29] Liu J Chen I Du Q Chua H Kwang J. The ORF3 protein of porcine circovirus type 2 is involved in viral pathogenesis in vivo. J Virol. (2006) 80:5065–73. 10.1128/JVI.80.10.5065-5073.2006 16641298 PMC1472074

[30] Kinsella CM Bart A Deijs M Broekhuizen P Kaczorowska J Jebbink MF. Entamoeba and Giardia parasites implicated as hosts of CRESS viruses. Nat Commun. (2020) 11:4620. 10.1038/s41467-020-18474-w 32934242 PMC7493932

