# Supplementary figures and images for: Feline Stool-Associated Circular DNA Virus (FeSCV) in Diarrheic Cats in China
Source: Front Vet Sci. 2021 Jun 16;8:694089. doi: 10.3389/fvets.2021.694089 (PMC8242157; doi:10.3389/fvets.2021.694089)

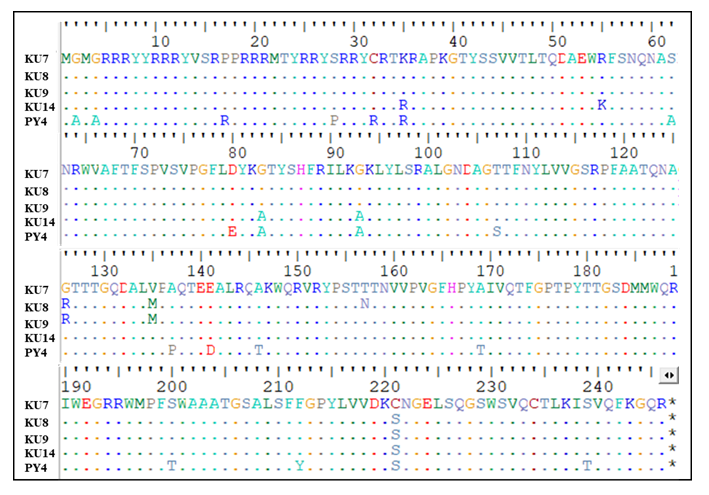

Supplement: Supplementary Figure 1 — Amino acid map of the FeSCV Cap protein. The ClustalW Multiole alignment method was used to analyze amino acid mutations in BioEdit (version 7.0.9.0). [file Image_1.TIFF]
